# Pensions for Nurses

**Published:** 1924-10

**Authors:** 


### PENSIONS FOR NURSES: WHAT IS BEING DONE.

(FROM A CORRESPONDENT.)

A recent discussion in the *Times* on pensions for nurses has aroused a good deal of attention among those interested in nurses and their activities. Everyone concerned would be glad of any scheme which would attain the desirable object of providing for nurses when past work. The difficulties which beset such a scheme, however, are so great that its realisation, at any rate in present conditions, is practically impossible. If every nurse at the end of her training remained either in the hospital where she was professionally educated or moved from that hospital to another and always remained in kindred institutions, there would be the possibility of arriving at some practicable way of making provision for her future when past work. The majority of nurses, however, do not remain in institutional work, but after leaving their training schools either join a co-operative society or work on their own account, obtaining their cases directly from doctors.

### No Help from Patients.

By whatever means they obtain their cases their position remains the same—they are nursing private patients. Whether the fees charged are heavy or not, whether or not they represent the true value of the work which the nurse is doing, is beside the point. It may be said, without fear of contradiction, that, whatever fees are charged, the average patient will think them high. Patients, or their friends, even object to paying the fivepence a week for stamping the cards under the National Health Insurance Acts. It is, therefore, unlikely in the highest degree that these same people will make any contribution towards the superannuation of the nurse. They have no interest in her beyond her professional services for the comparatively short time that she is in the house. When the National Health Insurance Act (1911) was in Bill form efforts were made to obtain the exemption of nurses, but it was said by those in authority that if the door was opened so as to let out any one profession there would be such a rush of others that it would be impossible ever to close it. However high our appreciation of the fact that the work of the nurse differs entirely from that of other professional women, it is difficult to find any particular reason why those in private practice should be treated differently from other working women—governesses, shorthand writers, musicians, etc., because after all they are giving their work for a certain wage. It is true that the work of the nurse is of a beneficial character, but, to be perfectly honest, it is not done for philanthropic reasons and the pay for the work is, as in other professions, the best that can be obtained, however bad that best may be.

### The Root of the Difficulty.

There are other reasons which militate against the success of a universal pension scheme for nurses, the chief difficulty being at the very outset. The majority of hospitals find it difficult to exist. Their committees are always busy thinking out new schemes by which to raise money to minister to the wants of the sick and suffering and to maintain the hospital generally. The unfortunate superintendent or secretary, or by whatever name he may be known, is continuously harassed by the bugbear of making ends meet and is therefore not over anxious to encourage any scheme which would mean a further claim on his budget.

### What the Hospitals might do.

Moreover hospital committees are too anxious to consider the wants of those for whom they minister, without much regard to those who do the ministering. If only committees would realise that they have a duty to their workers in the same way that the managers of great railways or of large commercial undertakings do, half the trouble would disappear, this half being the insurance of the worker for some measure of superannuation from the time she is qualified. If the worker moves on to another hospital or kindred institution the insurance could either lapse or be maintained by the nurse. On the other hand, should the worker remain in institutional work for the remainder of her nursing life the document—or by whatever name the policy was called—issued to the first hospital could be passed on to the hospital or institution to which she took her work and so at the end of her career she would be assured of some measure of superannuation. The only way to attain the object is for hospitals to come into line. They must all accept the principle that it is necessary to make provision for the superannuation of their workers, and this desirable end can only be achieved with the support and encouragement of the British Hospitals Association, a body which has done such good work for nurses as well as for hospitals.

### What is Being Done.

Meanwhile in the Royal National Pension Fund for Nurses, which has now been in existence for nearly forty years, nurses are provided with a ready means of helping themselves to pensions varying with the amount they are able to save. The chief object of the Fund—one of its founders was the late Sir Henry Burdett, the founder of the *Hospital and Health Review*—is to afford to nurses an absolutely safe means of providing, at the lowest possible cost to themselves, a certain income for their declining years. This object is carried out by receiving and investing such fixed periodical sums as those who join the fund can afford, by adding to the pensions all the profits arising from any source, and by supplementing those sums from a Donation Bonus Fund, created and maintained by persons interested in nurses and nursing institutions.

### Overseas Nursing Association.

The Overseas Nursing Association has issued its Annual Report for 1923-4. There has been an increasing demand for Nurses, and new candidates continue to apply. The number of Nurses at work during the year was 512, of whom 134 were employed as Private Nurses and in Hospitals not under Government, 8 in the Dominions, and 370 in Government Hospitals in the Crown Colonies.

